# Artificial dural regeneration matrix as a substitute for autologous tissue in indirect bypass in Moyamoya disease: Investigation of a rat model of chronic cerebral hypoperfusion

**DOI:** 10.1007/s10143-025-03185-x

**Published:** 2025-01-14

**Authors:** Koki Kameno, Yasuyuki Kaku, Yuki Ohmori, Yushin Takemoto, Ken Uekawa, Akitake Mukasa

**Affiliations:** 1 Department of Neurosurgery, Kumamoto University Hospital, 1-1-1 Honjo, 860-8556 Chuo-ku Kumamoto, Japan; 2 Division of Cerebrovascular and Cardiovascular Pathophysiology, Graduate School of Medical Sciences, Kumamoto University, Kumamoto, Japan

**Keywords:** Angiogenesis, Cerebral hypoperfusion, DuraGen, Indirect bypass, Moyamoya disease

## Abstract

Indirect bypass using autologous tissue is effective in Moyamoya disease, especially among pediatric patients. This study aimed to evaluate the effectiveness of indirect bypass using DuraGen (absorbable artificial dura mater composed of collagen matrix), as a substitute for autologous tissue in a rat model of chronic cerebral hypoperfusion. Male Wistar rats were subjected to bilateral internal carotid artery occlusion and divided into three groups: a control group without bypass surgery, a group wherein indirect bypass was performed using the temporalis muscle (encephalo-myo-synangiosis [EMS] group), and a group wherein DuraGen was used (Dura group). The ratio of the number of vascular endothelial cells, detected by antibodies to CD31 and glucose transporter type 1 (Glut-1), on the operative side to that on the non-operative side was measured and compared between the three groups. The ratio of CD31-positive cells was 1.50 ± 0.13 and 1.92 ± 0.29 in the EMS and Dura groups, respectively, and that of Glut-1-positive cells was 1.32 ± 0.10 and 1.53 ± 0.18 in the EMS and Dura groups, respectively. No significant difference was observed in the ratio of vascular endothelial cells on the bypass side between the EMS and Dura groups. Indirect bypass with DuraGen resulted in an increased ratio of vascular endothelial cells, equivalent to that of an indirect bypass with the temporalis muscle in a rat model. Thus, in an actual indirect bypass for patients with Moyamoya disease, the use of DuraGen may produce the same angiogenesis as using autologous tissue.

## Introduction

Moyamoya disease (MMD) is a steno-occlusive disease characterized by chronic progressive stenosis in the terminal portions of the bilateral internal carotid arteries and the compensatory development of collateral vessels, called ‘‘moyamoya vessels,’’ at the base of the brain [1]. Various methods of revascularization have been reported to be effective in preventing recurrent strokes [2–5]. Direct bypass with superficial temporal artery (STA)–middle cerebral artery (MCA) anastomosis is known to be effective in preventing recurrent stroke because it allows for early hemodynamic reconstruction [6]. However, this approach is sometimes challenging, particularly in children with fragile vessels. In contrast, indirect bypass, including encephalo-myo-synangiosis (EMS), encephalo-duro-arterio-synangiosis, and encephalo-duro-arterio-myo-synangiosis, are relatively easy to perform, although they take time to improve blood flow, and have been shown to be as effective as direct bypass in preventing recurrent strokes, especially in pediatric patients [7, 8].

As revascularization procedures use subcutaneous tissue, including the STA, temporal muscles, and galea, wound complications such as alopecia, skin necrosis, and infection often occur [9]. Previous studies have reported wound complication rates of 0.7%–31.7% [10, 11]. In patients with MMD, who generally have a good long-term prognosis, wound complications such as alopecia are chronic issues that should be avoided as they may interfere with the patient’s social life. Wound complications are caused by impairment of scalp microcirculation, not only due to the harvesting of the STA but also due to the involvement of subcutaneous tissues, including the galea and temporal muscles [9]. Therefore, we considered using artificial dura mater as an alternative tissue for indirect bypass. In particular, DuraGen^®^ (Integra LifeSciences, Princeton, NJ, USA) is an absorbable artificial dura mater composed of a collagen matrix, and its use in dural defects results in fibroblast infiltration and regeneration of dura-like tissue. Fibroblasts have been reported to play an important role in angiogenesis [12]. Therefore, we expected that fibroblast infiltrating DuraGen in hypoperfusion conditions such as MMD might also have a favorable effect on angiogenesis.

In this study, we hypothesized that DuraGen would be sufficiently effective in indirect bypass and prevent wound complications by sparing normal skin structures. To validate this hypothesis, we used a rat model of chronic cerebral hypoperfusion, subjected to bilateral internal carotid artery occlusion (BICAO) to determine whether indirect bypass with DuraGen induces angiogenesis comparable to that induced by indirect bypass with the temporalis muscle.

## Material and methods

### Animal and experimental group

All experiments were approved by the Committee for Laboratory Animal Care and Use at Kumamoto University. Male Wistar rats weighing 293.3–346.7 g (9–10 weeks old) were used. All rats were group-housed with two to three animals per cage and reared in a temperature-controlled (20 ± 2 ℃) and humidity-controlled (50%–60%) room under a 12-h light/dark cycle (8:00/20:00). All rats were subjected to BICAO prior to the indirect bypass procedure and divided into the following three experimental groups:

1. Control group (BICAO without indirect bypass; *n* = 6).
2. EMS group (BICAO with indirect bypass using the temporalis muscle; *n* = 7).
3. Dura group (BICAO with indirect bypass using DuraGen; *n* = 6).

### Bilateral internal carotid artery occlusion and indirect bypass

**Fig. 1:**
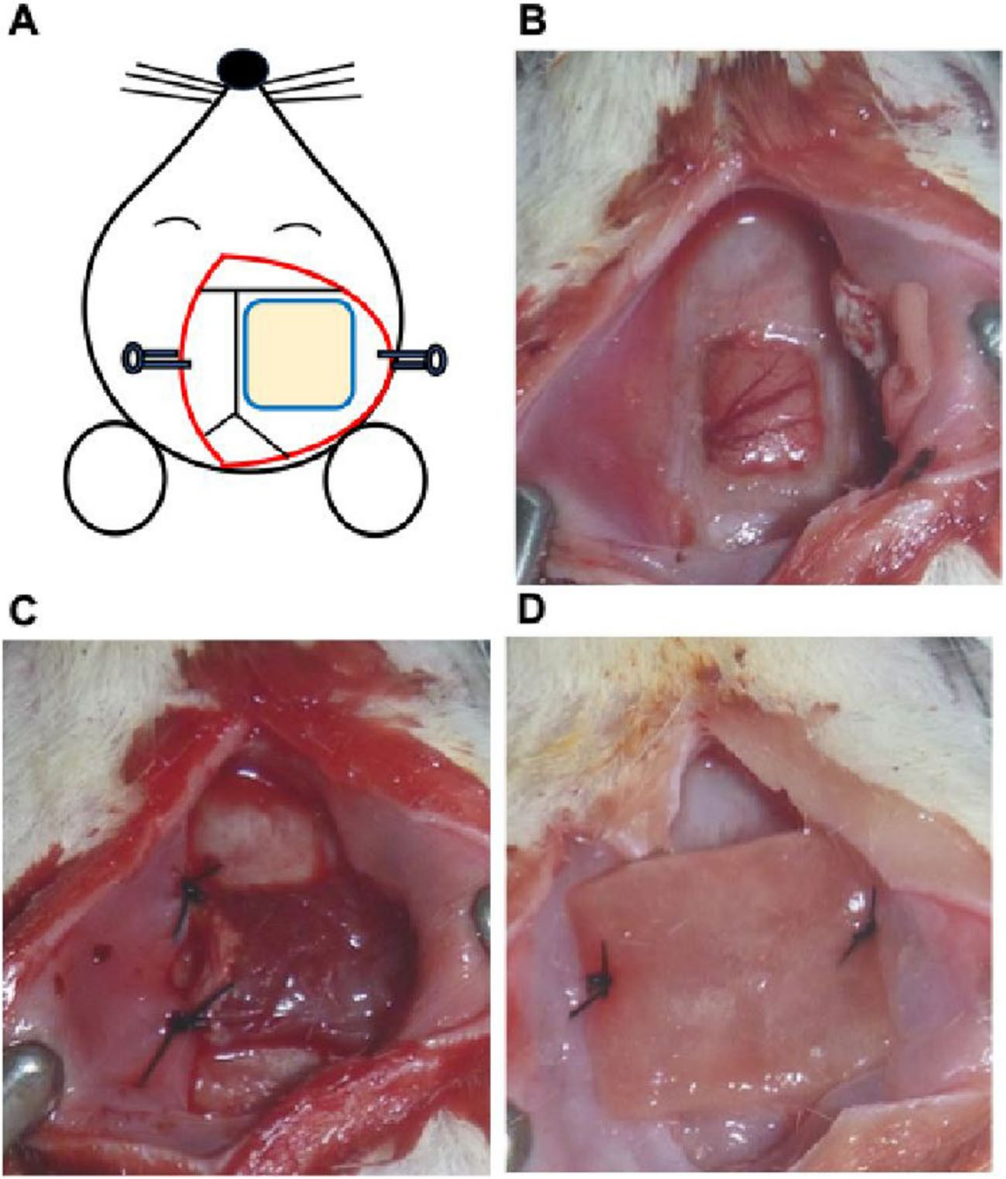
Illustration of craniotomy (**A**), surgical image after craniotomy (**B**), indirect bypass with the temporalis muscle (**C**), and indirect bypass with DuraGen (**D**)

All surgical procedures were performed under anesthesia with 2% isoflurane. BICAO was performed as previously described [13]. Briefly, bilateral internal carotid arteries were exposed via a ventral midline cervical incision and double ligated with 4–0 silk sutures. After BICAO, the rats were placed on a stereotactic head holder with the top of the skull positioned horizontally. A parieto-occipital midline skin incision was made, and the galea was peeled off the skull. A 7-mm^2^ craniectomy was performed carefully by drilling behind the coronal suture, 1 mm right lateral to the midline (Fig. 1A and B), as described in a previous study [13]. The dura was removed using microsurgical forceps and a microscope to avoid brain injury. In the EMS group, the brain surface was covered by temporal muscles (Fig. 1C). In contrast, in the Dura group, DuraGen was secured to the surrounding craniotomy using 4–0 nylon sutures to cover the brain surface (Fig. 1D). In the control group, to prevent angiogenesis due to inflammatory mechanisms, craniectomy was not performed. All rats were observed for 6 weeks after BICAO, with or without indirect bypass, and were then sacrificed.

### Histology

The rats were sacrificed 6 weeks after the indirect bypass, and brain tissues were collected as described previously [13]. Briefly, all rats were anesthetized with isoflurane. Subsequently, the rats were transcardially perfused with phosphate-buffered saline (PBS), followed by perfusion with 4% paraformaldehyde in PBS. The brain tissue with indirect bypass was carefully removed. Coronal sections with the indirect bypass tissue, measuring 5 mm, were obtained from each brain, fixed in 4% paraformaldehyde for 48 h, and embedded in paraffin. Coronal sections were sliced to 4-μm thickness. The sections were stained with hematoxylin and eosin for histological evaluation. Sections were photographed at 40 × magnification. The remaining sections were prepared and subjected to immunofluorescence staining.

Sections were immunostained with platelet-derived growth factor receptor alpha (PDGFR-α, 1:100, Santa Cruz Biotechnology, Inc, Dallas, TX, USA) for 1 h to detect fibroblasts between the indirect bypass tissue and brain cortex. Positive staining was detected using a detection kit (Histofine; Nichirei, Tokyo, Japan) by incubating the sections with 3,3′-diaminobenzidine chromogen (Dako North America, Inc., Carpinteria, CA, USA).

CD31 and glucose transporter type 1 (Glut-1) were used as markers of vascular endothelial cells and were evaluated through immunofluorescence staining. CD31 was incubated overnight with the primary antibody (1:50, Abcam 28,364, Abcam, Cambridge, UK) at 4 °C, and Glut-1 (1:10,000, Sigma-Aldrich Co., Burlington, MA, USA) was incubated for 1 h at 24 °C. CD31 and Glut-1 were then incubated with goat polyclonal anti-rabbit immunoglobulin G secondary antibody (Alexa Fluor 488, Abcam) for 1 h to identify cell immunoreactivity. After counterstaining with 4,6-diamidino-2-phenylindole for 24 h at 24 °C, three areas of the brain cortex under the indirect bypass and the contralateral cortex were visualized using fluorescent microscopy (BZ9000, Keyence Co., Osaka, Japan) at 200 × magnification. The number of CD31- or Glut-1–positive vascular endothelial cells was automatically measured. The effect of indirect bypass on angiogenesis was expressed as the ratio of the average number of vascular endothelial cells on the operative side to those on the non-operative side. The ratios were then compared among the three groups. Statistical analysis was performed using one-way analysis of variance.

## Results

### Histological examination

**Fig. 2:**
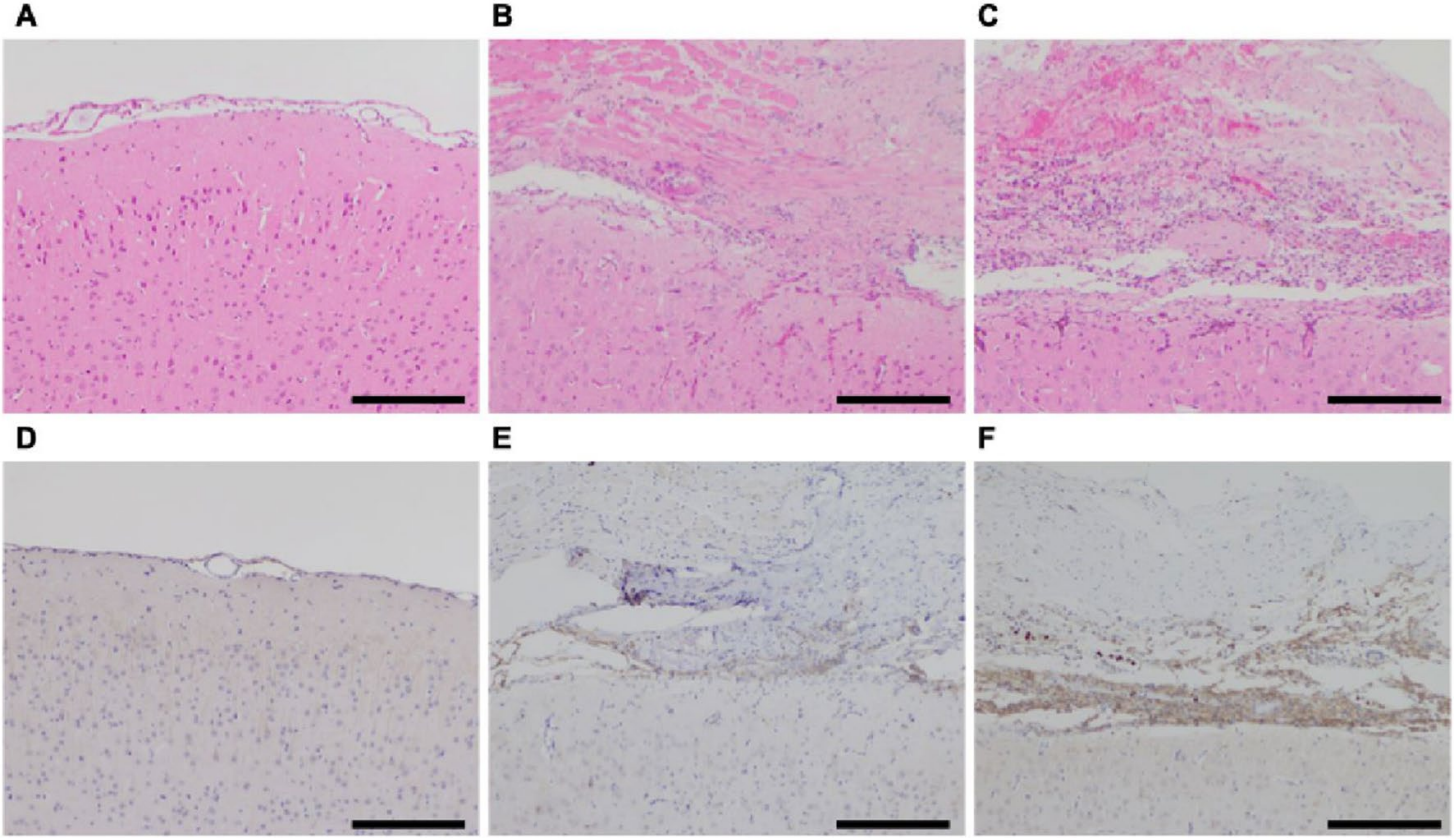
Hematoxylin and eosin staining of the coronal section in the control (**A**), EMS (**B**), and Dura groups (**C**). Immunohistochemical staining for platelet-derived growth factor receptor alpha at the brain surface in the control group (**D**) and at the tissue between the indirect bypass and cortex in the EMS (**E**) and the Dura groups (**F**). The scale bar represents 200 μm. EMS, encephalo-myo-synangiosis

In the control group, no transdural anastomosis was observed under chronic cerebral hypoperfusion alone (Fig. 2A). Conversely, adhesion between the brain surface and indirect bypass tissue was confirmed using hematoxylin and eosin staining in both the EMS and Dura groups (Fig. 2B and C). In the Dura group, cellular infiltration into the dura-like connective tissue, which DuraGen was replaced, was observed (Fig. 2C). Immunohistochemical staining showed that the infiltrated cells were PDGFR-α-positive and likely fibroblasts (Fig. 2F). PDGFR-α-positive cells between the brain cortex and indirect bypass tissue were more clearly stained in a representative case from the Dura group (Fig. 2E and F). In the control group, a few PDGFR-α-positive cells were observed in the dura mater (Fig. 2D).

**Fig. 3:**
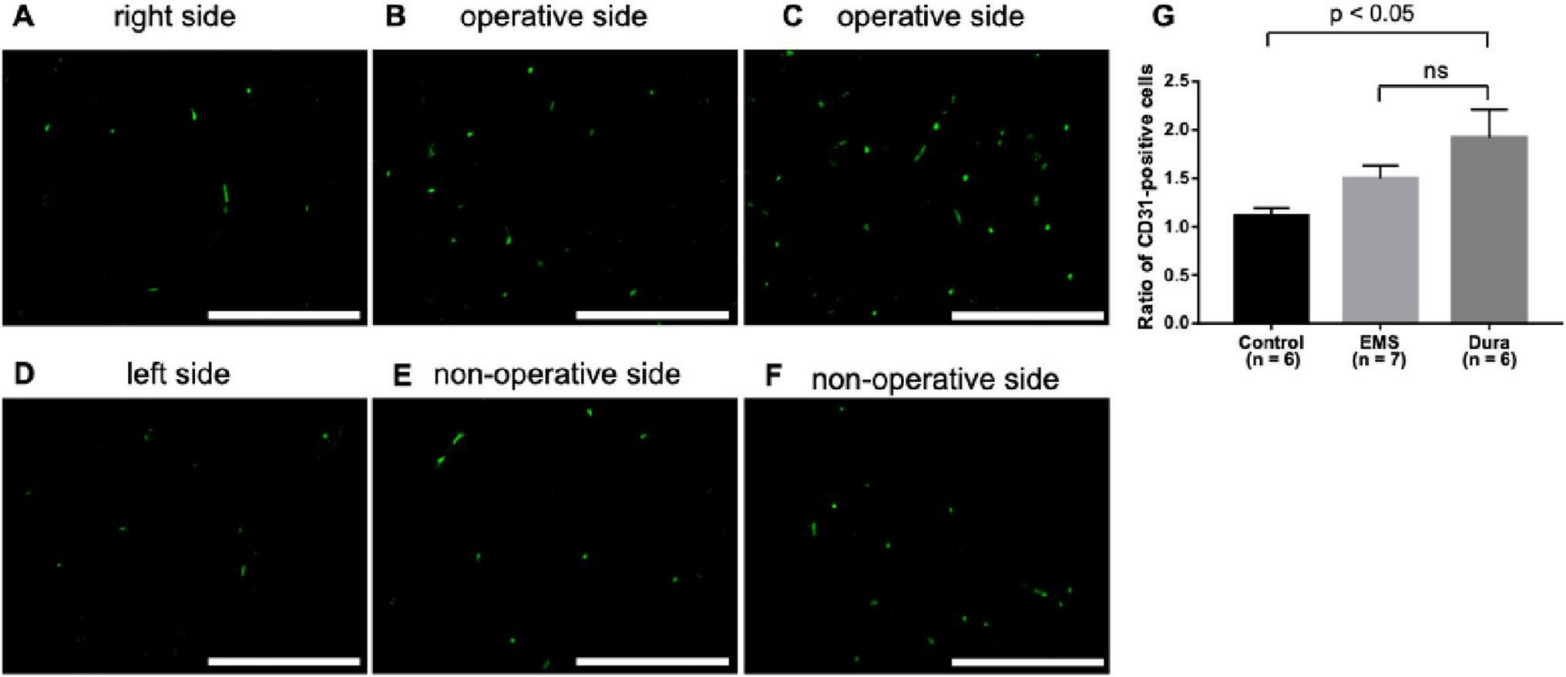
Photomicrographs of immunofluorescence using primary antibodies against CD31 in the cortex at the right side (**A**) and the left side (**D**) of the control group, the operative side (**B**) and the non-operative side (**E**) of the indirect bypass with the temporalis muscle and the operative side (**C**) and the non-operative side (**F**) of the indirect bypass with DuraGen. The scale bar represents 100 μm. The ratio of CD31-positive cells at the operative side is compared with those at the non-operative side (**G**)

**Fig. 4:**
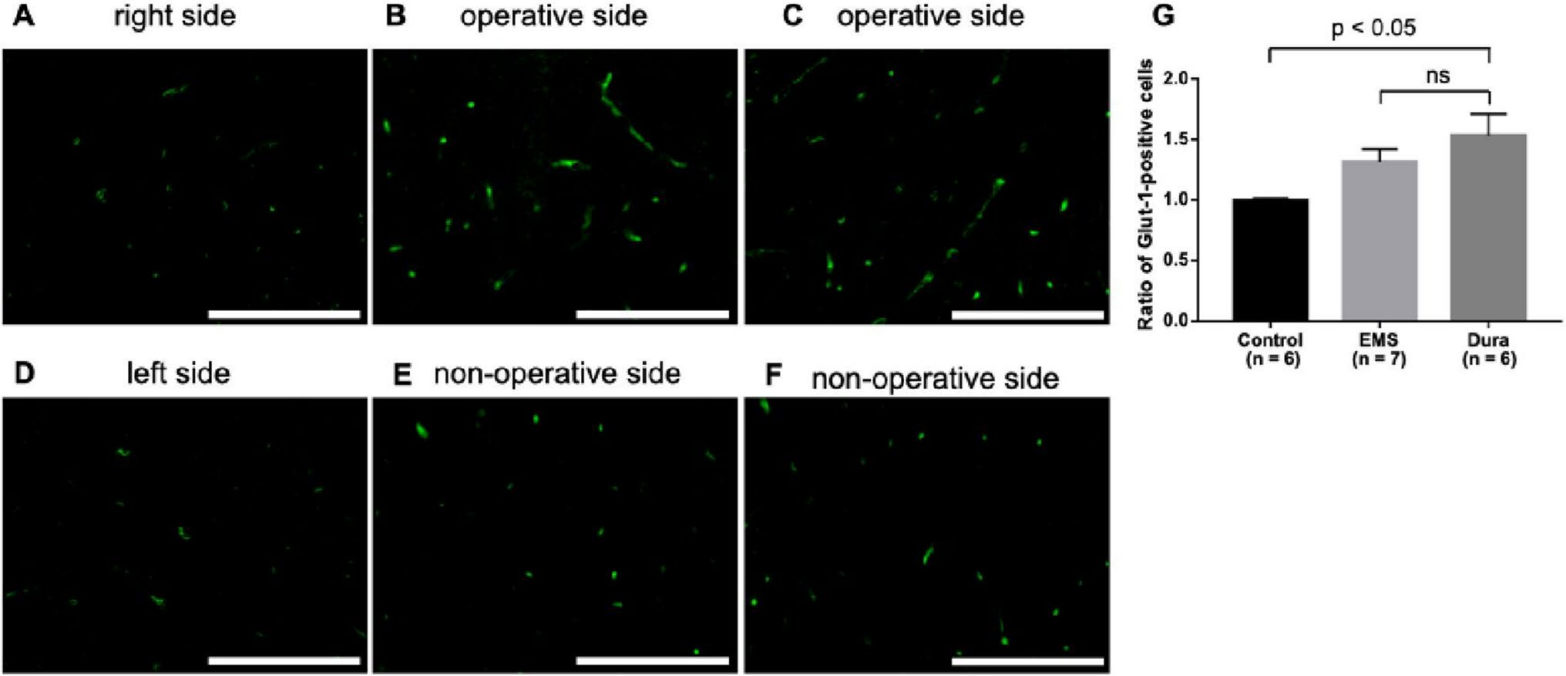
Photomicrographs of immunofluorescence using primary antibodies against Glut-1 in the cortex at the right side (**A**) and the left side (**D**) of the control group, the operative side (**B**) and the non-operative side (**E**) of the indirect bypass with the temporalis muscle, and at the operative side (**C**) and the non-operative side (**F**) of the indirect bypass with DuraGen. The scale bar represents 100 μm. The ratio of Glut-1–positive cells at the operative side is compared with those at the non-operative side (**G**) Glut-1, glucose transporter type-1

The ratios of CD31-positive cells were 1.11 ± 0.08, 1.50 ± 0.13, and 1.92 ± 0.29 in the control, EMS, and Dura groups, respectively (Fig. 3A–F). No significant difference was observed in the proportion of CD31-positive cells between the EMS and Dura groups (Fig. 3G). The Dura group showed a significant increase in the number of CD31-positive cells compared with the control group (Fig. 3G). Similar results were obtained for the number of vascular endothelial cells stained with Glut-1. The ratios of Glut-1-positive cells were 1.0 ± 0.02, 1.32 ± 0.10, and 1.53 ± 0.18 in the control, EMS, and Dura groups, respectively (Fig. 4A–F). No significant difference was observed in the proportion of Glut-1-positive cells between the EMS and Dura groups (Fig. 4G).

### Illustrative case of DuraGen use in a patient with MMD

**Fig. 5:**
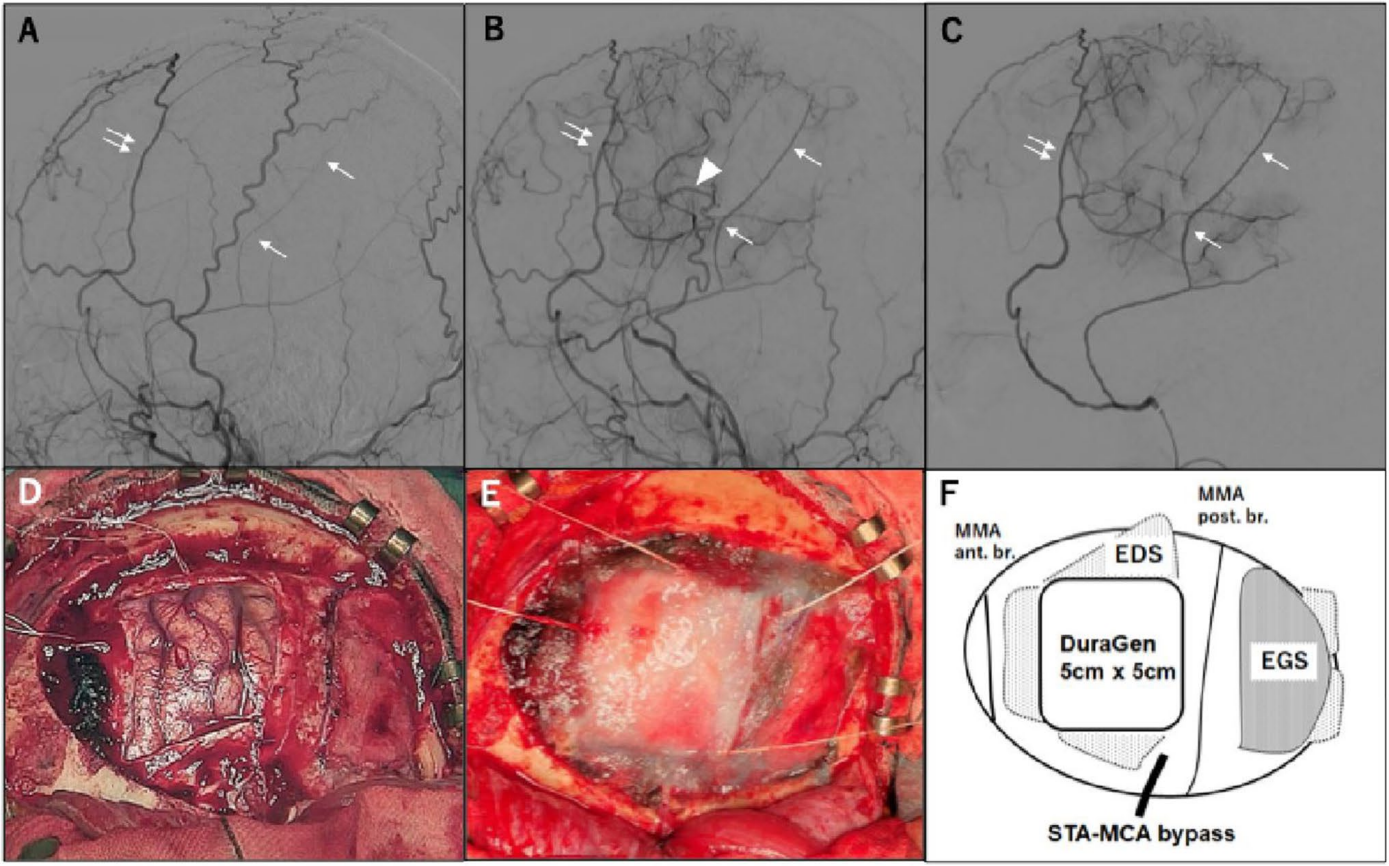
Preoperative external carotid angiography showing spontaneous collateral routes to the ACA via the MMA anterior branch (**A**). Postoperative external carotid angiography showing the development of surgical collaterals via the STA and MMA (**B**). The MMA posterior branch dilated owing to the blood supply. Superselective angiography of the MMA shows its contribution to the blood supply of the EDS (**C**). Intraoperative photograph during indirect bypass using DuraGen after direct bypass (**D**, **E**). Schema of the operation (**F**). Two branches of the MMA are preserved, and the dura mater is incised, followed by an indirect bypass of the EDS and EGS. DuraGen was used to repair the dura mater defect

A 32-year-old woman presented with loss of consciousness. Computed tomography revealed a cerebral hemorrhage in the left ventricle. Carotid angiography revealed severe stenotic lesions at the terminal portions of the bilateral internal carotid arteries and basal moyamoya vessels, leading to a definitive diagnosis of MMD. After 1 month of conservative treatment, the patient underwent STA-MCA bypass and an indirect bypass (encephalo-galeal-synangiosis [EGS]). DuraGen was used to treat dura mater defects. Carotid angiography 6 months after surgery revealed the development of surgical collaterals via the STA and the middle meningeal artery (MMA). Superselective angiography of the MMA revealed that it contributed to the blood supply of encephalo-duro-synangiosis (EDS). DuraGen itself did not induce angiogenesis, and the brain surface at the site where DuraGen was used for dural defects was supplied by marginal EDS (Fig. 5).

Doble arrows indicate the MMA anterior branch; arrows, MMA posterior branch; arrowhead, STA. ACA, anterior cerebral artery; EDS, encephalo-duro-synangiosis; EGS, encephalo-galeal-synangiosis; MMA, middle meningeal artery; STA, superficial temporal artery.

## Discussion

This study demonstrated that indirect bypass with DuraGen increased the ratio of vascular endothelial cells, making it equivalent to that of indirect bypass with the temporalis muscle in a rat model of chronic cerebral hypoperfusion.

This study aimed to determine whether DuraGen can be used as a substitute for the temporalis muscle during indirect bypass in MMD. Although experimental studies examining MMD generally use the bilateral common carotid artery occlusion model [14, 15], this study used the BICAO model. This was possible for two reasons:

1. The BICAO model is more similar to MMD than the bilateral common carotid artery occlusion model.
2. Blood supply from the external carotid artery was important because we sought to examine angiogenesis after indirect bypass, which creates transdural anastomosis via the external carotid artery.

Based on the aforementioned opinions, we previously reported that rats subjected to BICAO show a sufficient reduction in cerebral blood flow of approximately 50%–60% and that granulocyte colony-stimulating factor enhances revascularization in rats subjected to EMS after BICAO [13]. Therefore, the rat BICAO model was considered acceptable for simulating the effects of indirect bypass on MMD.

DuraGen is a sheet composed of collagen matrix that is infiltrated by fibroblasts and regenerates dura-like tissues [16, 17]. Similar to findings in a previous study [16], hematoxylin and eosin staining showed that DuraGen was replaced with connective tissue and infiltrated by a large number of PDGFR-α-positive cells. PDGFR-α is a fibroblast marker, and PDGFR-α–positive fibroblasts and collagen deposition are common in the arachnoid membranes of patients with MMD [18]. Hayashi et al. reported that PDGFR-α knockout mice showed reduced angiogenesis after EMS and decreased collagen deposition between the temporalis muscle and brain surface, indicating that PDGFR-α and collagen deposition induced by PDGFR-α signaling play an important role in angiogenesis following EMS [12]. These results indicate that angiogenesis may be induced in rats after BICAO by fibroblasts infiltrating the collagen sheet DuraGen, which serves as a scaffold.

Regarding angiogenesis, no significant difference was observed between indirect bypass using the temporalis muscle and DuraGen in the increased ratios of both CD31- and Glut-1-positive cells on the operative side. Furthermore, the Dura group showed a significant increase in the number of vascular endothelial cells on the bypass side compared to the control group. In a rat model of BICAO, indirect bypass using DuraGen showed a similar rate of increase in vascular endothelial cells compared to indirect bypass using the temporalis muscle.

The effects of revascularization for MMD are widely recognized [5, 6]. Indirect bypass using tissues such as the temporalis muscle and galea is effective, especially in pediatric patients [7, 8]. However, skin wound healing requires an optimal scalp microcirculation. In this study, we demonstrated that indirect bypass using DuraGen has the potential to induce angiogenesis comparable to that induced by indirect bypass using the temporalis muscle in a rat model of chronic cerebral hypoperfusion. However, in the presented case, it was unclear whether the observed angiogenesis developed from the DuraGen itself or from adjacent EDS or EGS. Therefore, in clinical practice, DuraGen may be considered a rescue material for dural defects in conjunction with EDS. However, the use of DuraGen preserves the temporalis muscle or galea, potentially reducing wound complications.

The present study has some limitations. First, we assessed the effect of indirect bypass on angiogenesis by comparing the number of vascular endothelial cells. The results showed that the increased ratio of vascular endothelial cells in the indirect bypass using DuraGen was similar to that in EMS. However, we could not detect the vessels penetrating the brain surface from DuraGen. Therefore, we were unable to demonstrate DuraGen-mediated angiogenesis directly. To confirm the effect of angiogenesis on indirect bypass, the control group did not be underwent craniectomy and dural incision to ensure that angiogenesis by any mechanism did not occur. Therefore, we have not been able to assess the extent to which craniectomy and dural incision affect angiogenesis on indirect bypass with temporal muscles or DuraGen. Further investigation comparing sham-operated brains which are underwent craniectomy and dural incision is needed to clarify the effect and mechanism of angiogenesis by DuraGen itself. Second, in this experimental study, indirect bypass was performed with craniectomy, unlike indirect bypass surgery in humans. Because the scalp is perfused with abundant blood flow, angiogenesis may have been induced by the scalp, which could serve as a source of angiogenesis owing to its large contact area with DuraGen. Whether angiogenesis can be induced after indirect bypass using DuraGen, even without contact with the scalp, requires further investigation. Third, the present study did not evaluate neurological symptoms in rats. Further studies are required to determine whether DuraGen provides sufficient revascularization to prevent hypoperfusion-induced neuropathy.

## Conclusion

In conclusion, indirect bypass using DuraGen resulted in an increased ratio of vascular endothelial cells equivalent to that of indirect bypass with the temporalis muscle in a rat model of chronic cerebral hypoperfusion. This result indicates that angiogenesis was induced by DuraGen, which was equivalent to that in the temporalis muscle. In an actual indirect bypass for patients with MMD, using DuraGen instead of subcutaneous tissue, including the temporalis muscle, may preserve the normal subcutaneous tissue layer, prevent wound healing failure and alopecia, and produce the same level of angiogenesis as conventional indirect bypass.

## Data Availability

No datasets were generated or analysed during the current study.

## References

[1] Ihara M, Yamamoto Y, Hattori Y et al (2022) Moyamoya disease: diagnosis and interventions. Lancet Neurol 21:747–758. 10.1016/S1474-4422(22)00165-X 35605621 10.1016/S1474-4422(22)00165-X

[2] Acker G, Fekonja L, Vajkoczy P (2018) Surgical management of Moyamoya disease. Stroke 49:476–482. 10.1161/STROKEAHA.117.018563 29343587 10.1161/STROKEAHA.117.018563

[3] Ozgur BM, Aryan HE, Levy ML (2006) Indirect revascularisation for paediatric Moyamoya disease: the EDAMS technique. J Clin Neurosci 13:105–108. 10.1016/j.jocn.2005.04.008 16410206 10.1016/j.jocn.2005.04.008

[4] Shirane R, Yoshida Y, Takahashi T, Yoshimoto T (1997) Assessment of encephalo-galeo-myo-synangiosis with dural pedicle insertion in childhood Moyamoya disease: characteristics of cerebral blood flow and oxygen metabolism. Clin Neurol Neurosurg 99(Suppl 2):S79–85. 10.1016/s0303-8467(97)00062-0 9409412 10.1016/s0303-8467(97)00062-0

[5] Li Q, Gao Y, Xin W et al (2019) Meta-analysis of prognosis of different treatments for symptomatic Moyamoya disease. World Neurosurg 127:354–361. 10.1016/j.wneu.2019.04.062 30995556 10.1016/j.wneu.2019.04.062

[6] Jeon JP, Kim JE, Cho WS, Bang JS, Son YJ, Oh CW (2018) Meta-analysis of the surgical outcomes of symptomatic Moyamoya disease in adults. J Neurosurg 128:793–799. 10.3171/2016.11.JNS161688 28474994 10.3171/2016.11.JNS161688

[7] Lee KS, Zhang JJY, Bhate S, Ganesan V, Thompson D, James G, Silva AHD (2023) Surgical revascularizations for pediatric moyamoya: a systematic review, meta-analysis, and meta-regression analysis. Childs Nerv Syst 39:1225–1243. 10.1007/s00381-023-05868-6 36752913 10.1007/s00381-023-05868-6 PMC10167165

[8] Piao J, Wu W, Yang Z, Yu J (2015) Research progress of Moyamoya disease in children. Int J Med Sci 12:566–575. 10.7150/ijms.11719 26180513 10.7150/ijms.11719 PMC4502061

[9] Acker G, Schlinkmann N, Fekonja L, Grünwald L, Hardt J, Czabanka M, Vajkoczy P (2019) Wound healing complications after revascularization for moyamoya vasculopathy with reference to different skin incisions. Neurosurg Focus 46:E12. 10.3171/2018.11.FOCUS18512 30717062 10.3171/2018.11.FOCUS18512

[10] Abla AA, Gandhoke G, Clark JC et al (2013) Surgical outcomes for moyamoya angiopathy at barrow neurological institute with comparison of adult indirect encephaloduroarteriosynangiosis bypass, adult direct superficial temporal artery-to-middle cerebral artery bypass, and pediatric bypass: 154 revascularization surgeries in 140 affected hemispheres. Neurosurgery 73:430–439. 10.1227/NEU.0000000000000017 23756739 10.1227/NEU.0000000000000017

[11] Chung Y, Lee SH, Choi SK (2017) Fundamental basis of scalp layering techniques to protect against wound infection: a comparative study between conventional and in-to-out dissection of the superficial temporal artery. World Neurosurg 97:304–311. 10.1016/j.wneu.2016.10.002 27742506 10.1016/j.wneu.2016.10.002

[12] Hayashi T, Yamamoto S, Hamashima T, Mori H, Sasahara M, Kuroda S (2020) Critical role of platelet-derived growth factor-alpha in angiogenesis after indirect bypass in a murine Moyamoya disease model. J Neurosurg 134:1535–1543. 10.3171/2020.3.JNS193273 32442967 10.3171/2020.3.JNS193273

[13] Ohmori Y, Morioka M, Kaku Y, Kawano T, Kuratsu J (2011) Granulocyte colony-stimulating factor enhances the angiogenetic effect of indirect bypass surgery for chronic cerebral hypoperfusion in a rat model*.* Neurosurgery 68:1372–1379; discussion 1379. 10.1227/NEU.0b013e31820c0289 10.1227/NEU.0b013e31820c0289 21273924

[14] Hiramatsu M, Hishikawa T, Tokunaga K et al (2017) Combined gene therapy with vascular endothelial growth factor plus apelin in a chronic cerebral hypoperfusion model in rats. J Neurosurg 127:679–686. 10.3171/2016.8.JNS16366 28009234 10.3171/2016.8.JNS16366

[15] Nishihiro S, Hishikawa T, Hiramatsu M et al (2019) High-mobility group Box-1-Induced angiogenesis after indirect bypass surgery in a chronic cerebral hypoperfusion model. NeuroMolecular Med 21:391–400. 10.1007/s12017-019-08541-x 31123914 10.1007/s12017-019-08541-x PMC6882763

[16] Khorasani L, Kapur RP, Lee C, Avellino AM (2008) Histological analysis of DuraGen in a human subject: case report. Clin Neuropathol 27:361–364. 10.5414/npp27361 18808069 10.5414/npp27361

[17] Haq I, Cruz-Almeida Y, Siqueira EB, Norenberg M, Green BA, Levi AD (2005) Postoperative fibrosis after surgical treatment of the porcine spinal cord: a comparison of dural substitutes. Invited submission from the Joint Section Meeting on Disorders of the Spine and Peripheral Nerves, March 2004. J Neurosurg Spine 2:50–54. 10.3171/spi.2005.2.1.0050 10.3171/spi.2005.2.1.0050 15658126

[18] Yamamoto S, Yamamoto S, Akai T, Sasahara M, Kuroda S (2022) Differentiation of fibroblasts into myofibroblasts in the arachnoid membrane of Moyamoya disease. Stroke 53:3465–3473. 10.1161/STROKEAHA.122.039961 36039752 10.1161/STROKEAHA.122.039961

